# Identifiability and model misspecification for modelling recurrent infections using routine health care data

**DOI:** 10.1093/aje/kwag138

**Published:** 2026-06-17

**Authors:** Ada W C Yan, Jennifer A Flegg, Jeanne Rini Poespoprodjo, Nicholas M Douglas, Ric N Price, Angela Devine, David J Price, Rebecca H Chisholm

**Affiliations:** Department of Mathematical and Physical Sciences, La Trobe University, Melbourne, Australia; Department of Infectious Disease, Imperial College London, United Kingdom; Department of Mathematical and Geospatial Sciences, RMIT University, Melbourne, Australia; School of Mathematics and Statistics, The University of Melbourne, Melbourne, Australia; Yayasan Pengembangan Kesehatan Dan Masyarakat Papua (YPKMP), Timika, Indonesia; Mimika General Hospital, Timika, Indonesia; Menzies School of Health Research, Charles Darwin University, Darwin, Australia; Centre for Child Health-pro, Faculty of Medicine, Public Health and Nursing Universitas Gadjah Mada, Yogyakarta, Indonesia; Menzies School of Health Research, Charles Darwin University, Darwin, Australia; Department of Medicine, University of Otago, Christchurch, New Zealand; Department of Infectious Diseases, Christchurch Hospital, Te Whatu Ora, Christchurch, New Zealand; Menzies School of Health Research, Charles Darwin University, Darwin, Australia; Nuffield Department of Clinical Medicine, Centre for Tropical Medicine and Global Health, University of Oxford, Oxford, United Kingdom; Mahidol-Oxford Tropical Medicine Research Unit (MORU), Faculty of Tropical Medicine, Mahidol University, Bangkok, Thailand; Menzies School of Health Research, Charles Darwin University, Darwin, Australia; Melbourne School of Population and Global Health, The University of Melbourne, Melbourne, Australia; Melbourne School of Population and Global Health, The University of Melbourne, Melbourne, Australia; Department of Infectious Diseases, The University of Melbourne, at the Peter Doherty Institute for Infection and Immunity, Melbourne, Australia; Department of Mathematical and Physical Sciences, La Trobe University, Melbourne, Australia; Melbourne School of Population and Global Health, The University of Melbourne, Melbourne, Australia

**Keywords:** recurrent infections, electronic health records, identifiability, mechanistic modelling, malaria

## Abstract

For infectious diseases where people can experience multiple infections during their lifetime, the time between observed infections in individuals (or “time to recurrence”) can provide valuable information on infection and transmission dynamics. Routinely collected data, such as electronic health records, are a potential source of time to recurrence data. However, they are challenging to analyse because patients can drop out of the data set in a way that is not visible to the data collection process. Standard epidemiological approaches, such as parametric survival analysis with imputation, cannot be applied to such data. In this study, we explored the feasibility of interrogating routinely collected time to recurrence data by calibrating mechanistic transmission models with explicit dropout mechanisms. We identified model structures and parameter regimes where the method could precisely and accurately estimate important epidemiological quantities. Application of our method to real data of malaria infections routinely collected in Papua, Indonesia, was able to estimate the forces of infection for different malaria species, the rate of dropout and recrudescence for *Plasmodium falciparum*, and the probability of treatment success. Our method has the potential to increase the value of existing and new data sets for informing public health research.

## Introduction

For infectious diseases where clearance does not confer lifelong immunity, times between individuals’ observed infections (or “time to recurrence”) can provide information on the dynamics of infection and transmission. For example, recurrent malaria infections can occur due to re-infection, recrudescence (rebound in parasitemia due to treatment failure) or relapse (reactivation of hypnozoites). These different aetiologies result in a complex distribution of the time to recurrence.

Previous malaria studies have applied parametric survival analysis [8] to clinical trial data to estimate the rate of relapse of *Plasmodium vivax* infections due to dormant hypnozoites [3, 21]. Other studies have interrogated clinical trial data by calibrating mechanistic transmission models to the distribution of time to recurrence, enabling detailed study of the effects of treatments on recurrence [6, 17, 23]

An important advantage of using clinical trial data is that the active follow-up captures all recurrences. Missed recurrences would lead to longer observed times between recurrences, biasing parameter estimates. However, clinical trial data may not be available; sample sizes may be small; or clinical efficacy may be hypothesised to differ from real-world effectiveness, for example if real-world adherence to a treatment regimen is poor. In such cases, observational study data and routinely collected data (such as electronic health records or data collected as part of a surveillance system) must be used to study the natural history of infection [1, 7, 18, 24, 25].

Routinely collected data poses a number of challenges. Individuals may permanently drop out of the data set due to a change in health care practitioner or domicile. Alternatively, individuals may temporarily drop out, for example due to inconsistent health care seeking behaviour (not always seeking health care when symptoms arise, or seeking care from different practitioners). Observational studies can face similar challenges, particularly in remote or nomadic communities where individuals may often be absent at follow-up [16]. However, in observational studies, loss to follow-up is recorded, which allows the use of standard imputation methods for survival analysis [15]. Dropout is not visible for routinely collected data and so standard imputation methods cannot be directly applied. In this study, we investigate whether calibrating mechanistic transmission models with explicit dropout mechanisms is a feasible approach to interrogating routinely collected data on infection. We then illustrate this method’s utility with routinely collected time to recurrence data for patients with *Plasmodium falciparum* and *P. vivax* malaria infection in Papua, Indonesia.

## Methods

Routinely collected data are not usually collected to calibrate mechanistic mathematical models. Therefore, it is necessary to conduct identifiability analyses to quantify the information we can expect to extract from routinely collected data. These analyses establish how accurately and precisely we can estimate various parameters from the data [2].

Here, we conduct structural and practical identifiability studies for five simplified mechanistic transmission models of increasing complexity that either explicitly incorporate or ignore permanent or temporary dropout of individuals from data collection. Practical identifiability is assessed using simulation-estimation studies conducted with Bayesian inference methods. For simplicity, in all models we assume that the population is at an endemic equilibrium, allowing us to impose a constant rate of new infections (the force of infection), *λ *.

Then, we describe how we investigate which parameters are identifiable from time to re-infection data and examine the effect of model misspecification on parameter estimation, where the model used to generate the data is different from the model used to estimate model parameters.

Lastly, we describe the routinely collected time to recurrence data for patients with malaria infection in Papua, Indonesia, and a model that we use to estimate parameters related to *P. falciparum* infection dynamics for these data.

### Infectious disease transmission and observation models with independent recurrences

We consider six models with increasing complexity. First, we consider a simple model with a constant re-infection rate and a perfect observation process (Model 1). Then, we consider different forms of dropping out from data collection (Models 2–4). Model 5 adds complexity in the infection process, including a period of treatment, time to seeking treatment, and asymptomatic re-infection. Model 6 includes the possibility of treatment failure and infection with different parasite species.

**Fig. 1.**
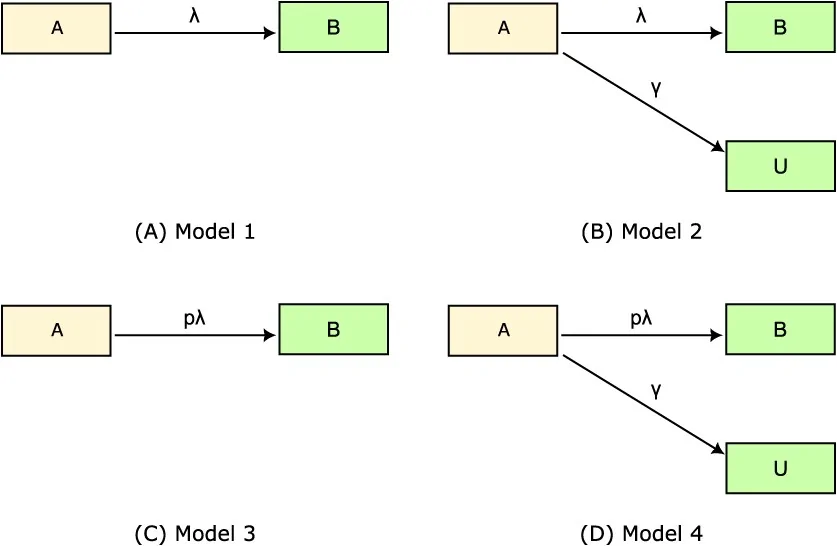
The four models considered in the study where we assume recurrent infections are independent of each other.

We start by considering a simple model (Model 1, Figure 1A), which consists of only two compartments. Individuals enter the model in the *A* compartment upon observation of their first infection. They transition to the *B* compartment when they become re-infected and observed again in the data, at a rate *λ *.

Adding permanent dropout from data collection results in Model 2 (Figure 1B). The *U* (unobserved) compartment represents individuals who have dropped out of the data collection process. Individuals start in *A* and either move to *B* at rate *λ *, or move to *U* at the dropout rate *γ *.

In Model 3 (Figure 1C), individuals do not permanently drop out, but each time they become re-infected, there is a probability *p* that they are recorded in the data (where *p < 1* due to eg, asymptomatic infection). Individuals start in *A* and move to *B* at a rate *pλ *. We assume that if an infection is not recorded, it does not affect the rate of the next re-infection; this assumption will be relaxed in Model 5.

Model 4 (Figure 1D) combines permanent and temporary dropout. We formulate Models 1–4 as continuous-time Markov chains (CTMCs). The states and transitions, as well as the Kolmogorov forward equations associated with the models, are provided in the Appendix S1, Tables S1–S2, Equations S1–S5.

### Infectious disease transmission and observation models with dependent recurrences

In Models 1–4 we assumed that an individual’s first infection and any subsequent unrecorded infections (including asymptomatic infections) do not affect the rate at which they have a second observed infection. However, if one infection must be cleared before another can occur, the time to recurrence distribution will change.

**Fig. 2.**
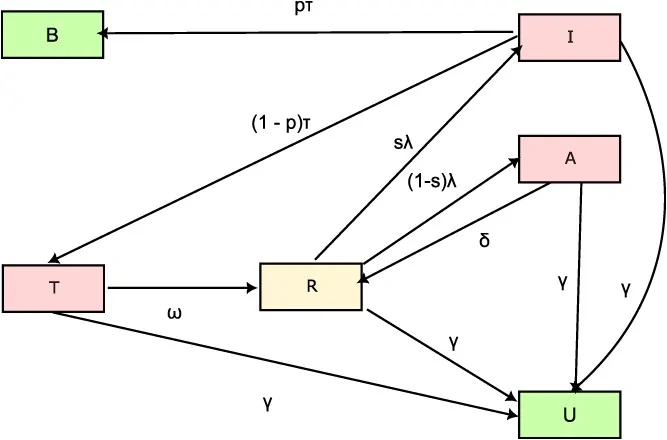
Model 5, where an ongoing infection excludes the possibility of a second infection.

To understand parameter identifiability when we account for this additional complexity, we consider Model 5 (Figure 2, Appendix S1, Equation S6). All individuals now start in the *T* (treated) compartment, and recover at a rate *ω *. Recovered individuals (*R*) become re-infected at a rate *λ *. Re-infections are symptomatic with probability *s*; these individuals transition to *I* (symptomatic re-infection). Otherwise, they transition to *A* (asymptomatic re-infection). Symptomatic infections are treated at a rate *τ *. There is a probability *p* that this treatment is observed in the data, and individuals transition to *B*, the “second observation” compartment as before. Otherwise, individuals transition back to *T*. Individuals with an asymptomatic infection (*A*) recover at a rate *δ *. Permanent dropout can occur from all compartments except *B* at a rate *γ *, leading to the unobserved compartment *U*.

All of these models assume a constant force of infection, such that the effects of seasonality, outbreaks, control measures, or changing immunity/behaviour are absent.

### Identifiability analysis

Identifiability can refer to structural identifiability, where parameters can be estimated accurately given perfect data [4], or practical identifiability, where parameters can be estimated accurately given realistic data [2, 12].

To establish the structural identifiability of Models 1–5, we used the Julia package *StructuralIdentifiability.jl* [10], and assumed that the distribution of observed times to recurrence is directly observed. Briefly, *StructuralIdentifiability.jl* uses the input-output approach to assess structural identifiability. It uses symbolic computation to eliminate state variables and derive a minimal set of relations between model parameters, inputs, and observed outputs. Then, it checks whether each parameter is in the differential field generated by the observed outputs and their derivatives, that is, whether each parameter can be expressed as a function of combinations of parameters that appear in the minimal set of relations.

To establish the practical identifiability of model parameters, we conducted simulation-estimation studies. For Models 1 and 2, we varied *1/λ * between 0.1 and 10 years, and *1/γ * (where applicable) between 0.5 and 10 years, using 56 combinations of the two parameters in total. For model 5, we used Latin hypercube sampling (implemented in *lhs* 1.2.0 [5] in R 4.4.0 [19]) to draw 100 parameter sets spanning the prior distribution specified in Appendix S1, Table S3. For these models, we then simulated data for *N = 1000* individuals for a study duration of *T = 10 × 365.25* days for each parameter set. We conducted these simulations using the continuous-time Markov chain version of the models (Appendix S1). Having simulated data across a range of viable parameter values, we then performed inference to re-estimate these parameters using Bayesian inference, using the No U-Turn Sampling implemented in *rstan* 2.32.6 [22]. Uniform priors were used to constrain parameter values to biologically reasonable ranges. The identifiability of Models 3 and 4 was derived from Models 1 and 2. Code to reproduce the analysis can be found at https://github.com/ada-w-yan/routinedatamalaria.git

To quantify the accuracy of parameter estimation, for each parameter set, the percentage error for each parameter value was calculated as $100(\hat{y} - y)/y$, where *y* is the true value of the model parameter and $\hat{y}$ is the median of the marginal posterior distribution.

### Model misspecification

We examined the effects of model misspecification by fitting Model 1 to data generated by Model 2, then quantifying the accuracy of parameter estimation as above. To address the limitation of Models 1–5 not capturing seasonality, we generate data from a model similar to Model 1, but where the force of infection varies seasonally (Model 1s, Supplementary Material). We then fit Model 1 to the generated data, and compare the value of *λ * fitted with the misspecified model to the true parameter value. Analysis of the structural and practical identifiability of Model 1s itself is beyond the study scope; more advanced mathematical techniques are required due to the time-dependent re-infection rate.

We also used posterior predictive checks to assess the effects of model misspecification. For each sample of parameter values in the joint posterior distribution, we simulated data for *N* individuals. The data from these *N* individuals was used to generate a cumulative risk curve using *fitdistr* 1.2.1 [9]. The median and 95% CI of the cumulative risk curve was computed across samples of the posterior distribution, and compared to the cumulative risk curve generated from the data.

### Real data

**Fig. 3.**
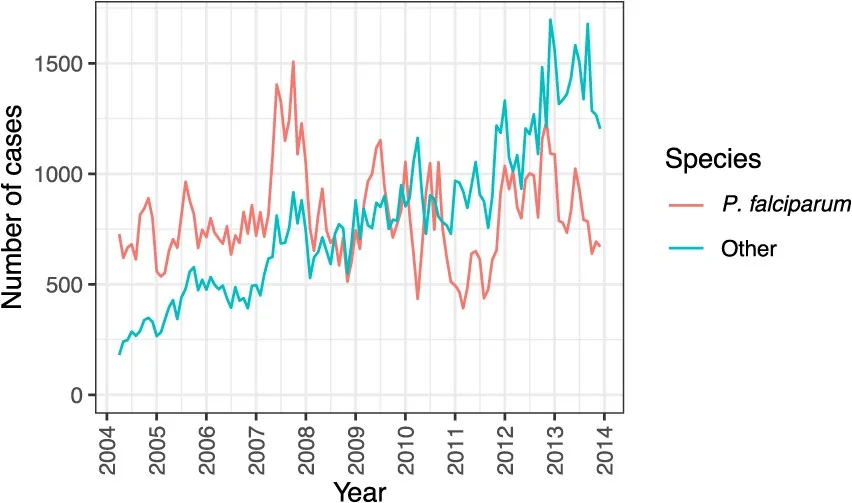
The number of *P. falciparum* episodes and malaria episodes with a species other than *P. falciparum* over time in the dataset, aggregated monthly.

The dataset has been described previously by Douglas *et al.* [11]. Briefly, patients presenting to the Rumah Sakit Mitra Masyarakat (RSMM) between 2004 and 2013 were routinely recorded in a Q-Pro database by hospital administrators. Each patient was assigned a unique identifier. For all patients diagnosed with malaria, the species of infection and the treatment given, as well as demographic data, were recorded. In data post-processing, multiple presentations within 14 days were combined into a single malaria episode. Between January 2004 and December 2013, a total of 186 869 malaria episodes were recorded for 68 530 individuals. Figure 3 shows the number of *P. falciparum* episodes and malaria episodes with a species other than *P. falciparum* over time, aggregated monthly.

To calculate the time to recurrence for patients initially presenting with *P. falciparum*, we filtered the dataset for all *P. falciparum* monoinfection episodes treated with either dihydroartemisinin–piperaquine (DHP) or quinine. These two treatments were chosen due to their large sample size in the data, and because their effectiveness has been well-characterised in previous studies. In the Indonesian context, recurrence is both more common and occurs more quickly for quinine compared to DHP [11, 13, 14, 20]. We could test whether our model replicates these known results.

For each first episode of malaria, the subsequent episode due to any malaria species in the same individual was identified using the patient’s unique identification number. If the individual did not have a further episode, we considered the second episode to be censored, with the censoring date being the end of the study (31/12/2013). The time to recurrence was calculated as the difference in the presentation dates for the two episodes.

### Model for real data

To capture the dynamic processes in the RSMM data requires more complexity than Models 1–5. First, a second episode of *P. falciparum* malaria could be due to re-infection or recrudescence. Reinfection arises after a patient is inoculated by a mosquito with the same or a different species of parasite. Recrudescence is due to rebound of parasitemia following partial or incomplete treatment of the first episode of malaria.

**Fig. 4.**
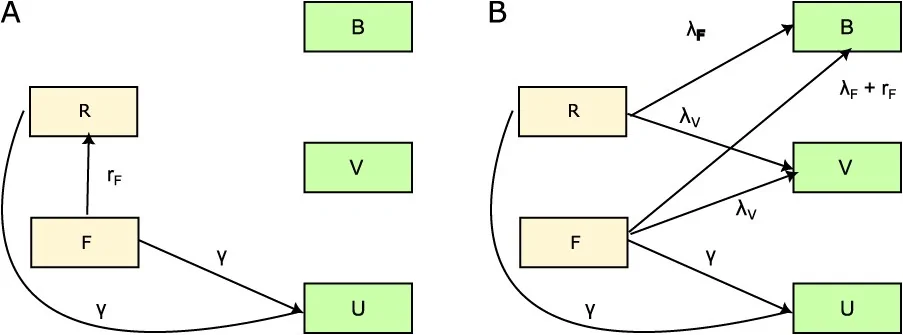
Compartmental diagram for Model 6 used for the case study, (A) during the first 14 days after the first observed *P. falciparum* infection, and (B) thereafter.

To capture recrudescence, we included a treatment failure compartment (*F*) in Model 6 (Figure 4). Individuals start in the *R* compartment if their treatment was successful, and in *F* otherwise. $p_{D}$ is the probability of treatment success, equal to the initial proportion of individuals in *R*. Individuals in *R* can become re-infected with *P. falciparum* at a rate $\lambda _{F}$. Individuals in *F* can recrudesce at a rate $r_{F}$, or become re-infected at the same rate $\lambda _{F}$. All individuals can drop out of the study at a rate *γ *.

Another complication is that re-infection can be with the same species of malaria parasite (*P. falciparum*), or a different species (such as *P. vivax*). If the second episode was with *P. falciparum*, then we cannot distinguish whether it resulted from re-infection or recrudescence. If the second episode is with a different species, then it cannot be from recrudescence. Although our primary objective is to understand *P. falciparum* dynamics, we cannot exclude episodes of re-infection from other species, because they are competing events affecting the dynamics of *P. falciparum*.

We captured re-infection with a species other than *P. falciparum* by including a compartment *V*. Individuals become re-infected with a species other than *P. falciparum* at a rate $\lambda _{V}$, and enter the compartment *V*. We interpret $\lambda _{F}$, $\lambda _{V}$, and $r_{F}$ as the rates of observed symptomatic re-infection with *P. falciparum*, observed symptomatic re-infection with any other species, and observed recrudescence.

Finally, any new infections or recrudescences within a 14-day time period were censored to match the data structure.

## Results

### Structural and practical identifiability analysis

#### The rate of permanent dropout, but not temporary dropout, can be inferred from time to re-infection data

**Fig. 5.**
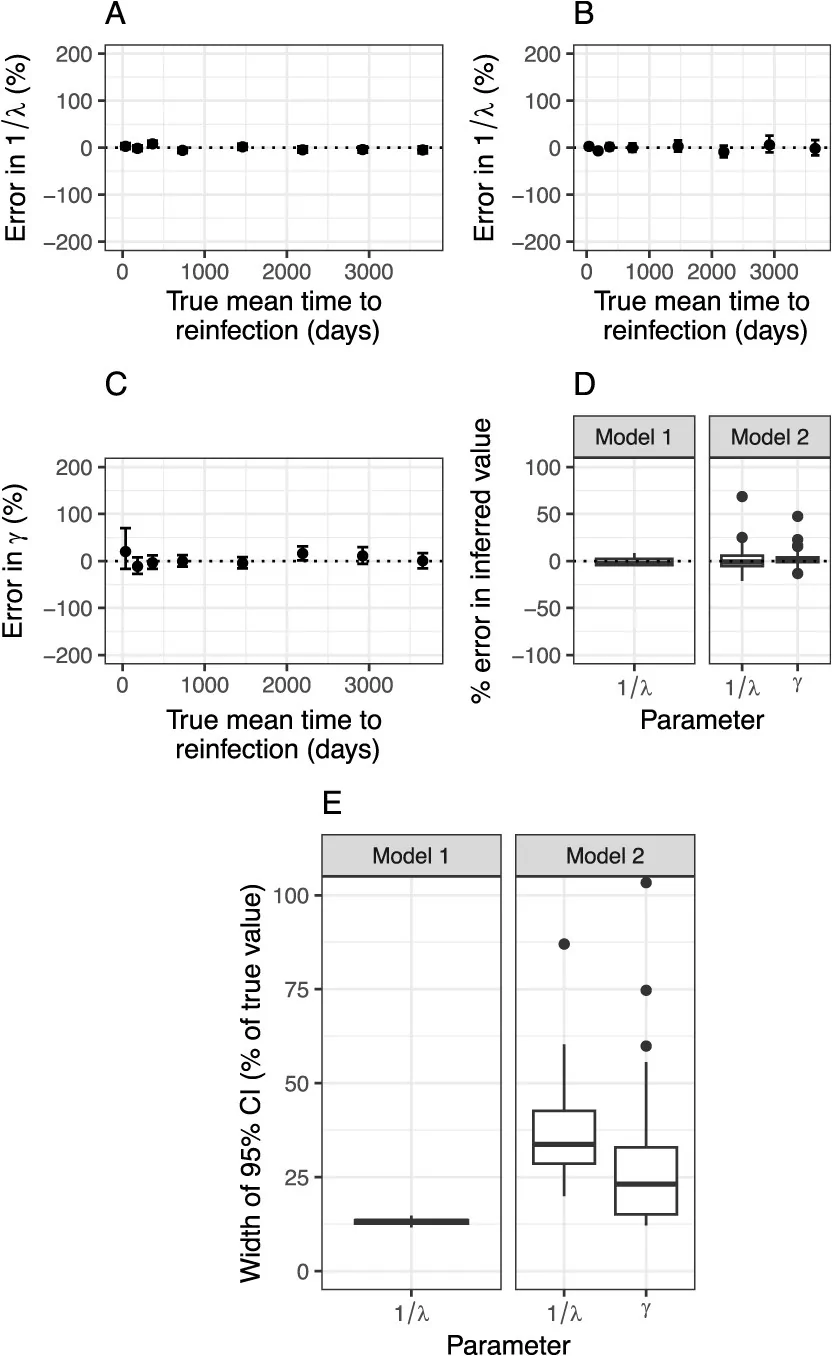
(A) Median and 95% CI for the percentage error in estimates of *1/λ * for Model 1, for different true values of *1/λ * (x-axis). (B) Median and 95% credible intervals (CI) for the percentage error in estimates of *1/λ * for Model 2, for different true values of *1/λ * (x-axis); true *1/γ * = 4 years. (C) Median and 95% credible intervals (CI) for the percentage error in estimates of *γ * for Model 2, for different true values of *1/γ * (x-axis); true *1/λ * = 4 years. (D) Boxplots for the percentage error in the inferred model parameters when fitting Models 1 and 2 to data generated using the same models. (E) Boxplots for the widths of the 95% CIs calculated using the marginal posterior distribution, expressed as a percentage of the true parameter values, when fitting Models 1 and 2 to data generated using the same models.

The practical identifiability assessment for Models 1–5 revealed that rate parameters related to infection, recovery and permanent dropout could be estimated precisely and accurately. Figures 5A–C and Appendix S1, Figure S1 show, for a subset of the 56 simulated parameter sets for Models 1–2, the median and 95% credible intervals (CI) for the percentage error in parameter estimates. Parameter estimation remained accurate and precise across the ranges of the true model parameters. Figure 5D combines these errors into a single boxplot for each parameter. For Model 1, all parameter sets tested resulted in < 10% error in *1/λ *. For Model 2, 88% of parameter sets resulted in < 10% error in *1/λ *, and 71% resulted in < 10% error in *γ *. Figure 5E shows the widths of the 95% CIs calculated using the marginal posterior distribution for each parameter, as a percentage of the true values; the 56 widths obtained across the parameter sets are combined into a single boxplot for each parameter. For Model 1, 75% of parameter sets resulted in the 95% CI width for *1/λ * being less than 14% of the true value. For Model 2, 75% of parameter sets resulted in the 95% CI width for *1/λ * being less than 33% of the true value, and the 95% CI width for *γ * being less than 43% of the true value. Thus, parameter estimates were both precise and accurate. In Models 3 and 4, the observation probability *p* and force of infection *λ * only appear as a product, so only their product (the rate of observed infections) is identifiable.

#### Model parameters associated with dependence between re-infections are unidentifiable

**Fig. 6.**
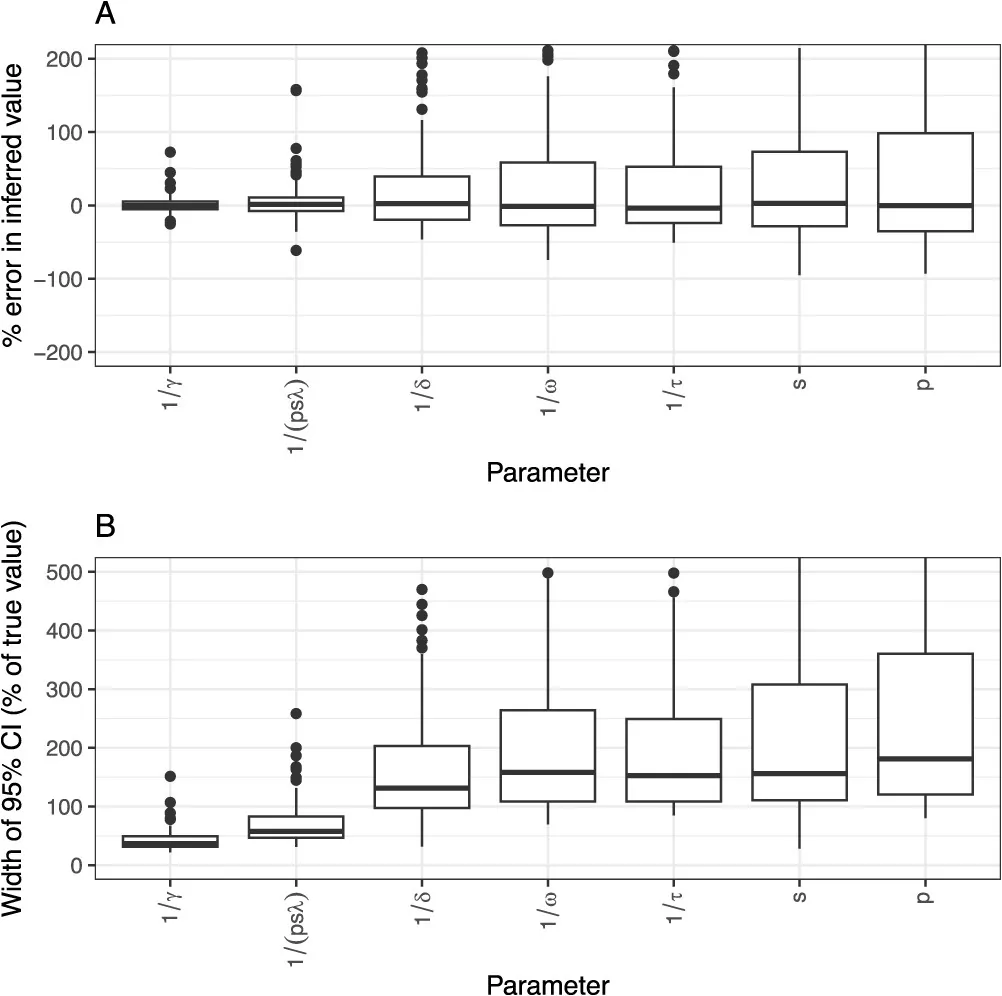
(A) Boxplots for the percentage error in the inferred model parameters when fitting Model 5 to data generated using the same model. (B) Boxplots for the widths of the 95% CIs calculated using the marginal posterior distribution, expressed as a percentage of the true parameter values, when fitting Model 5 to data generated using the same model. Appendix S1, Figure S3 shows the same figures without truncated axes.

For Model 5, only *p s λ * (the rate of an observed symptomatic infection) and *γ * were practically identifiable (Figure 6 and Appendix S1, Figure S2). This was because the rates *ω *, *τ *, and *δ * were much faster than the rates *λ * and *γ *. The time to event is a sum of exponentially distributed variables; the rates of the faster variables are not detectable in the multiphasic distribution.

For the parameters other than *p s λ * and *γ *, the errors for the parameter estimates spanned the prior distribution (Figure 6 and Appendix S1, Figures S2–S3). The absence of strong correlations in the joint posterior distribution for the unidentifiable parameters (Appendix S1, Figure S4) suggested that combinations of these parameters are also not identifiable.

One option is to fix the unidentifiable model parameters *p*, *s*, *1/ω *, *1/τ *, and *1/δ * at the mean values of their prior distributions, and fitting *1/(p s λ )* and *γ * only. Another option is to fit a simpler model (Model 2) to the data generated by Model 5. We fit the parameters *λ * and *γ *. We note that Model 5 reduces to Model 2 as $\omega , \tau , \delta \to \infty $ and *λ * is substituted for *psλ *. However, when $\omega , \tau $, and * δ * are finite, there is no parameter set for the full model that analytically reduces to the simpler model.

**Fig. 7.**
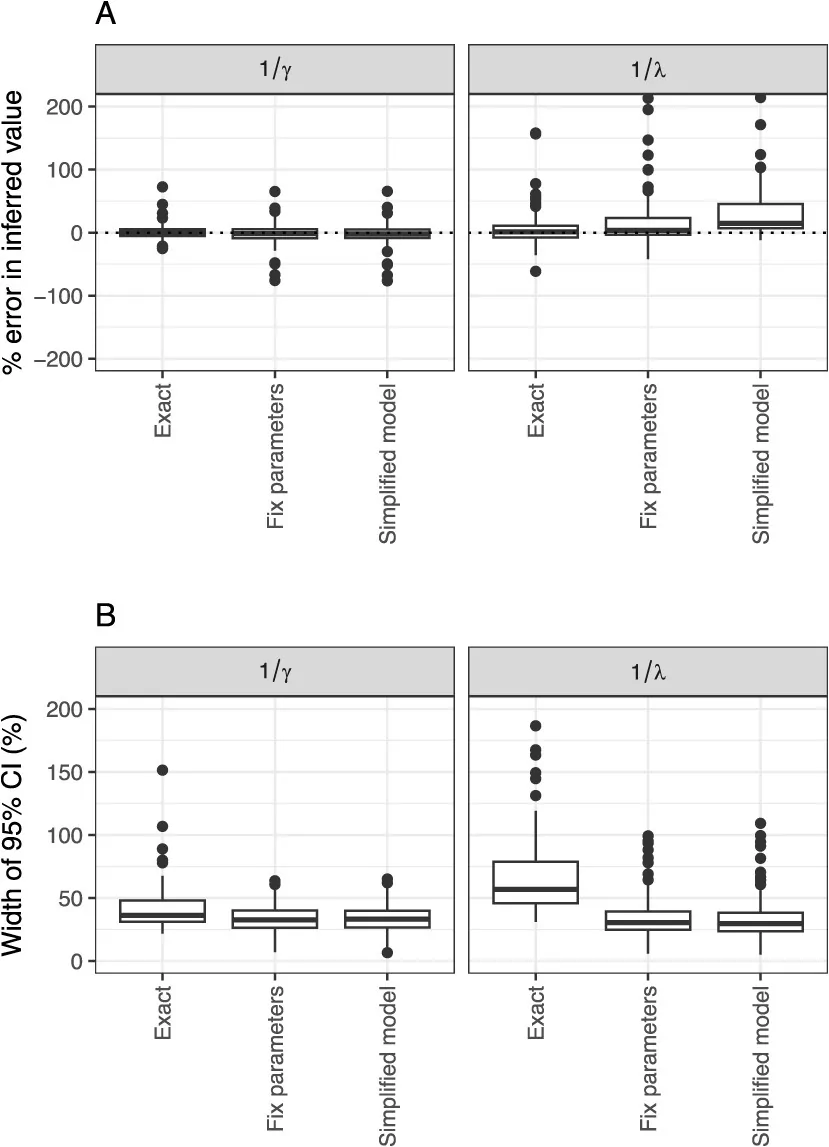
Boxplots for the percentage error and relative width of the 95% CI for *1/γ * and *1/λ *, using three model fitting methods on the data generated by Model 5. Some of the boxplots are truncated for readability; the full version is shown in Appendix S1, Figure S5.

Figure 7A and Appendix S1, Figure S5 show boxplots for the percentage error in *1/γ * and the mean time to observed symptomatic infection *1/(psλ )* when fitting all parameters in Model 5 (“exact”) or fixing model parameters (“fix parameters”); *1/λ * when fitting Model 2 instead (“simplified model”). All methods estimated *1/γ * equally well. For the mean time to observed symptomatic infection, fixing *p*, *s*, *1/ω *, *1/τ *, and *1/δ * at the mean values of their prior distributions resulted in slightly smaller errors than fitting a simpler model, but all methods performed well overall. Figure 7B shows boxplots for the 95% CI widths calculated using the marginal posterior distribution, expressed as a percentage of the true parameter values. The 95% CI width was similar for all methods when estimating *1/γ *, but was narrower when estimating *1/λ * and model parameters were fixed or the model was simplified.

The structural identifiability analyses mostly agreed with the practical identifiability results (Table 1). All model parameters were structurally identifiable for Models 1 and 2; for Model 3, the product *p λ * was identifiable; for Model 4, the products *p λ * and *γ * were identifiable. For Model 5, all parameters were structurally identifiable, but they were not all practically identifiable.

**Table 1:** Identifiable parameters for each model.

Model	Parameters	Structurally identifiable parameters	Practically identifiable parameters
1	*λ *	*λ *	*λ *
2	*λ * , *γ *	*λ * , *γ *	*λ * , *γ *
3	*p* , *λ *	*p λ *	*p λ *
4	*p* , *λ *, *γ *	*p λ * , *γ *	*p λ * , *γ *
5	*p* , *s*, *λ *, *γ *, *τ *, *ω *, *δ *	*p* , *s*, *λ *, *γ *, *τ *, *ω *, *δ *	*p s λ * , *γ *

### Model misspecification

#### Ignoring permanent dropout, but not temporary dropout, leads to inaccurate model predictions

We explored the effect of neglecting some modes of dropout on parameter identifiability in Models 1–4. By inspection, fitting Model 1 to data generated by Model 3, or Model 2 to data generated by Model 4 results in the inferred value of *λ * being equal to the true value of *p λ *. In these cases, *λ * should be interpreted as the rate of symptomatic infection rather than the rate of infection overall. Also by inspection, fitting Model 1 to data generated by Model 2 is equivalent to fitting Model 3 to data generated by Model 4, if *pλ * is substituted for *λ *.

**Fig. 8.**
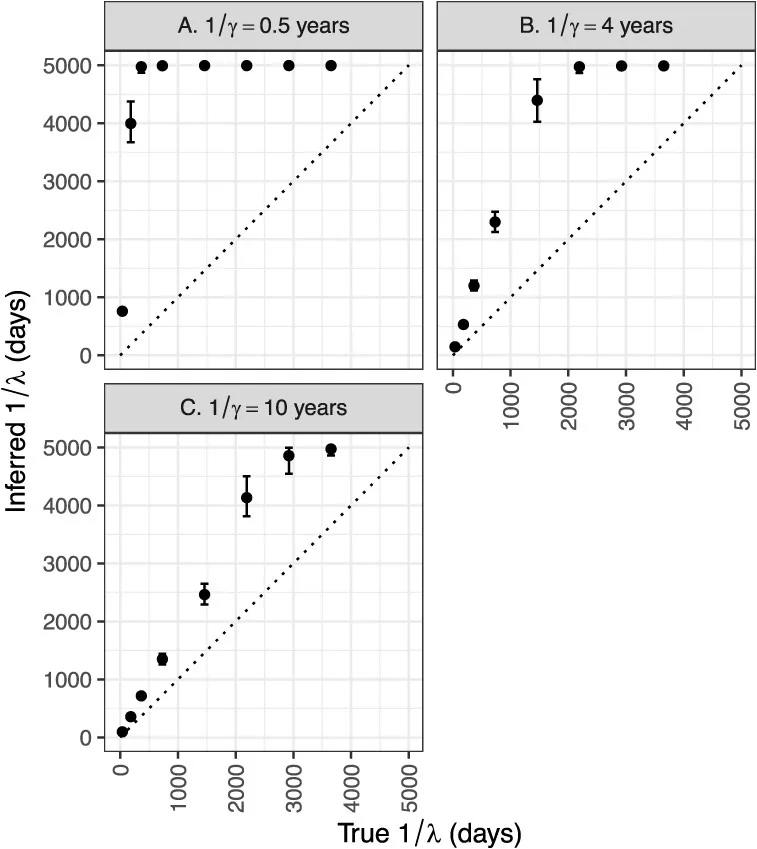
Median and 95% CI for estimates of *1/λ * when fitting Model 1 to data generated using Model 2, for different true values of *1/λ * in days (x-axis) and *1/γ * in years (subplots). The dotted line indicates *y = x*, that is an accurate estimate. The figure bounds are the bounds of the prior distribution.

Figure 8 shows the median and 95% CI for estimates of *1/λ * when fitting Model 1 to data generated using Model 2, for different true values of *1/λ * and *1/γ *. As expected, ignoring dropout led to a longer inferred mean time to re-infection, because if an individual does not have an observed re-infection, it is attributed to a lack of re-infection rather than dropout. This discrepancy increased with a higher dropout rate. Thus overly simplified models were not sufficient to handle data of this type.

The mis-estimation was apparent visually when looking at posterior predictions. Appendix S1, Figure S6 shows the 95% CI of cumulative risk curves simulated over the posterior distribution of *λ * obtained by fitting Model 1 to data generated using Model 2, for two different sets of parameter values. Saturation is apparent in the data but not the model predictions, and the model predictions cross over the data.

We could also detect the model misspecification using model selection. For each of the parameter sets, we used leave-one-out cross-validation (Appendix S1) to compare the predictive errors between Models 1 and 2 when they were fitted to data generated by Model 2. For all parameter sets, Model 2 was favoured statistically over Model 1, showing that we could detect model misspecification.

#### Ignoring seasonality only biases parameter estimates for a small range of true parameter values

**Fig. 9.**
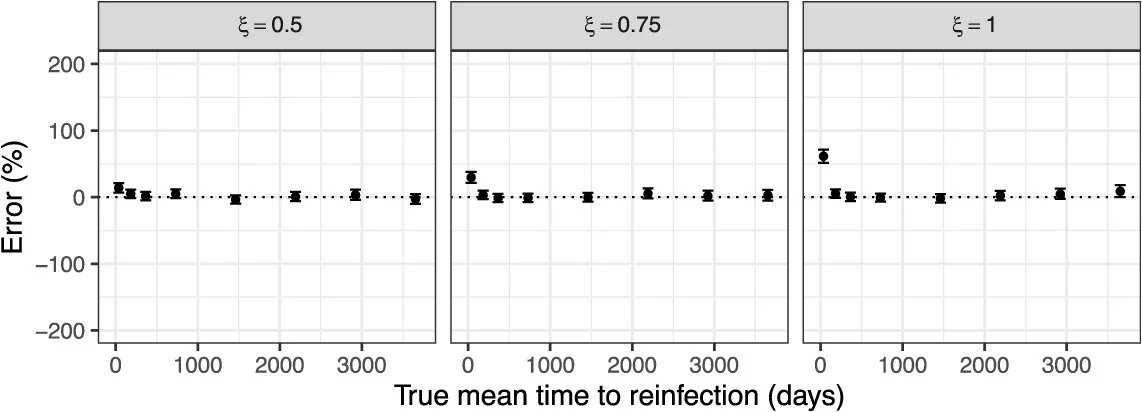
Median and 95% CI for the error in estimates of *1/λ * when fitting Model 1 to data generated using Model 1s, for different true values of *1/λ * in days (x-axis) and seasonality parameter *ξ * (subplots).

Figure 9 shows the median and 95% CI for the error in estimates of *1/λ * when fitting Model 1 to data generated using the same model but with seasonality in the force of infection (Model 1s), for different true values of *1/λ * in days (x-axis) and seasonality parameter *ξ * (subplots). Seasonality only introduced bias in the estimate of the mean time to re-infection *1/λ * when there was greater than a 50% difference between the peak force of infection and the mean force of infection (*ξ> 0.5*), and the true mean time to re-infection was less than half a year.

### Case study

**Fig. 10.**
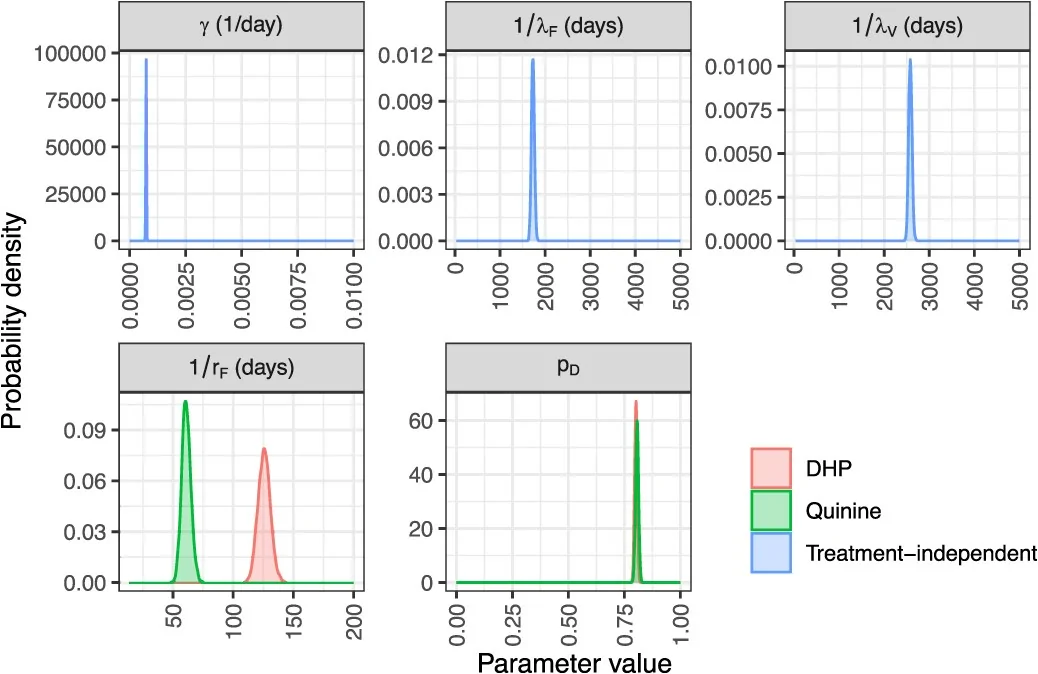
Posterior distributions for each parameter and treatment.

Fitting Model 6 (Figure 4) to the RSMM data resulted in precise estimates for all parameters (Figure 10). The probability of treatment success ($p_{D}$) was inferred to be similar for quinine (posterior median [95% Credible Interval], 0.80 [0.78, 0.81]) and DHP (0.81 [0.80. 0.83]), but the mean time to recrudescence ($1/r_{F}$) was much shorter for quinine (65 [57, 73] days) than DHP (121 [112, 133] days), as expected. As we used real data, the true values of these parameters, and thus the accuracy of parameter estimates, are unknown. Some of the model assumptions, such as the constant force of infection, are approximate and could affect the accuracy of parameter estimation. However, the model can provide rough estimates and general insights into the effectiveness of treatment.

We tested the assumption that the forces of infection from *P. falciparum* ($\lambda _{F}$), species other than *P. falciparum* ($\lambda _{V}$), and the dropout rate (*γ *) were treatment-independent, by fitting Model 6 separately to data from individuals first treated with DHP versus quinine (Appendix S1, Figure S7). The dropout rate *γ * was similar between treatments as expected (6.18 [5.90, 6.48]) $\times 10^{-4}$ per day for quinine, (8.50 [8.21, 8.78]) $\times 10^{-4}$ per day for DHP). The mean time to observed symptomatic infection for *P. falciparum*, $1/\lambda _{F}$, was also similar between treatments (1926 [1818, 2039] days for quinine, 1556 [1485, 1633] days for DHP). However, the mean time to observed symptomatic infection for a species other than *P. falciparum*, $1/\lambda _{V}$, was different between treatments (3698 [3493, 3915] days for quinine, 2194 [2123, 2265] days for DHP).

**Fig. 11.**
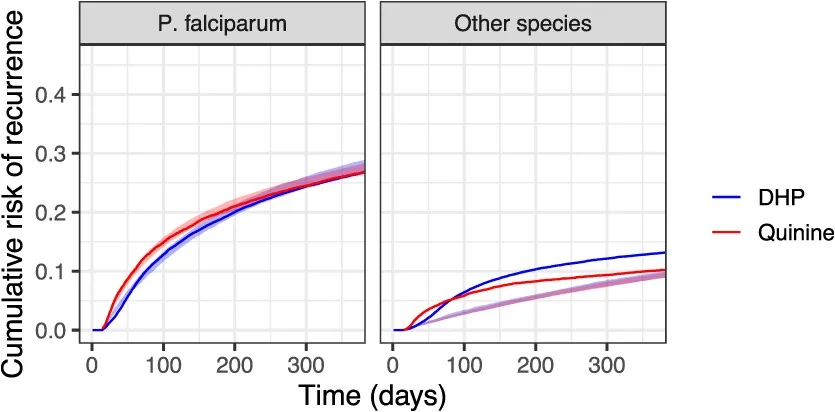
Data (dark lines), and 95% CI for posterior predictive distribution (lighter areas), for the cumulative risk of recurrence in the first year of the data collection with *P. falciparum* (left) or a different species (right), when the first infection is with *P. falciparum* and treated with (red) quinine (blue) DHP. Cumulative risk curves for the duration of the study are shown in Appendix S1, Figure S8A.

Comparing the posterior predictive distribution of the cumulative risk of recurrence for each species with that generated from the data (Figure 11 and Appendix S1, Figure S8A), the model predictions for recurrence with *P. falciparum* matched the data well.

In both the data and the model predictions, *P. falciparum* recurrence occurred more quickly for quinine than DHP. For recurrence with other species (primarily *P. vivax*), the model underpredicted the risk of recurrence. This was likely because the model did not account for *P. vivax* relapse. The same cumulative risk of recurrence was predicted for species other than *P. falciparum* regardless of the treatment for the first infection. This was because the force of infection was the driving factor for the rate of a second infection with a different species, and the force of infection was assumed to be the same between treatments. When the quinine and DHP data were fitted separately, the cumulative risks for infection with a species other than *P. falciparum* were predicted to be different between treatments, but still underestimated (Appendix S1, Figure S8B).

## Discussion

In this study, we used simulation-estimation studies and routinely collected malaria data to show that individual-level health records can be used to characterise the distribution of time to recurrence and to estimate key epidemiological parameters.

Simulation-estimation studies showed that for simple models, the force of infection and the rate of permanent dropout were estimated precisely and accurately. When each recurrence had an observation probability of less than 1, only the product of the observation probability and the force of infection was identifiable. If data were generated under a model with permanent dropout but fitted with a model without dropout, this misspecification was evident in posterior predictive checks and model selection criteria, suggesting that such checks can guide whether dropout should be included. By contrast, omitting seasonality when the true force of infection was seasonal had little impact on parameter estimates.

For a more complex model where recurrence cannot occur while there is an ongoing infection, model parameters were no longer identifiable. Fixing the values of the unidentifiable parameters or fitting a simpler model where the time to recurrence is independent of the dynamics of the first infection were both better approaches.

We then applied the methods to malaria data from Papua, Indonesia. We inferred the forces of infection with *Plasmodium falciparum* and with other species, the dropout rate, the recrudescence rate for *P. falciparum*, and the probability of treatment success for initial infections treated with quinine or DHP. When the model was fitted separately to quinine and DHP data, the treatments had different estimates of $\lambda _{V}$, the rate of observed symptomatic infection with non-falciparum species. This rate is implicitly the product of the underlying force of infection, the probability of symptomatic infection, and the probability of observation given symptoms. The force of infection should not depend on the antimalarial drug. However, a 2006 policy change led to quinine being prescribed mainly before 2006 and DHP after 2006 [11]. Figure 3 shows that the number of *P. falciparum* cases stayed constant throughout this period, while the number of cases due to other species increased. Thus, differences in $\lambda _{V}$ may reflect temporal variation in transmission as well as potential differences in symptom probability or care-seeking behaviour.

Previous studies [3, 6, 17, 21, 23] have estimated the force of infection from time to recurrence data from clinical trials or observational studies rather than routinely collected data. Our results indicate that limitations such as unknown dropout, which occur in routinely collected data, can be accommodated in mechanistic models. Consequently, both dropout and force of infection can be estimated reliably from routine surveillance if the mechanistic transmission and observation models adequately capture the epidemiological and data collection processes.

The aforementioned studies also did not explicitly address asymptomatic infections and their effect on the ability to estimate model parameters. We have shown that the proportion of asymptomatic infection and the rate of recovery from asymptomatic infection cannot be estimated from data on time between observed cases. Instead of including asymptomatic infection explicitly, we should estimate the rate of overall infection and bear in mind that this estimated rate is that of symptomatic infection.

This study has several limitations. For example, we assumed an exponential distribution for time to reinfection in the absence of competing processes, whereas other distributions might be more realistic, and we treated the probability of symptomatic reinfection and related parameters as age-independent. Instead of modelling dropout explicitly, one could right-censor follow-up after a fixed duration. To avoid within-individual correlation we restricted analysis to the first pair of recorded cases for each individual; because individuals were not necessarily infection-naive, this first recorded infection could itself be a reinfection, introducing unmodelled heterogeneity that could be captured by richer compartmental structures.

We assumed a constant force of infection corresponding to endemic equilibrium; in settings far from equilibrium the force of infection will vary over time. Future work will extend these methods to time-varying forces of infection and integrate them with approaches based on incidence time series.

We have shown that routinely collected data at the individual level can be used to estimate important parameters in simple mechanistic models of disease transmission, which has the potential to increase the value of existing and new data sets for informing public health research.
